# Selections and Abstracts

**Published:** 1898-11

**Authors:** 


### SELECTIONS AND ABSTRACTS.

### Should We Drink at Our Meals?

In an interesting and somewhat historical article, Dr. C. A. Ewald, of Berlin (*Zeitschrift für Kranhenpflege, Medical Record*), discusses this mooted question at some length. He considers soup, because of its small percentage of nourishing material, merely as fluid; he states that, aside from what is directly taken as drink, much fluid reaches the stomach during a meal, through the sauces and from the water percentage (both natural and by cooking) of the meats, vegetables, etc. Most persons feel the necessity of adding more fluid to the meal by drinking either ordinary water, carbonated waters, or alcoholic beverages. The more one eats, generally the more one drinks, and the greatest eaters are the greatest drinkers. If drink be prohibited, the amount eaten is less; indeed, on the above very greatly depends the secret of the "Schweinger cure" for obesity. It is a well-known fact that if the appetite is weak and the mind and nerves are somewhat relaxed, a drink of water will excite the appetite and stimulate both brain and nerves; and this is due directly to the fluid and not to the alcohol contained, for we find these facts the same in abstainers. The more fluid in the way of drink is added to the gastric juice, the greater is the quantity secreted, hence the greater the tax upon the gastric glands. Under normal circumstances, however, the stomach without detriment accommodates itself to a range of large quantities of fluid. Ewald says that much of the fluid passes into the intestines, another portion is absorbed; hence there never is in the normal stomach a stagnation of large quantities of liquid. The widely accepted belief that alcoholic fluids (not taken to the point of toxicity) retard digestion does not seem to be borne out by the recent experiments of Chittenden. Not even whisky and brandy seem to retard digestion. The proteolytic power of the pancreatic juice is uninfluenced by small quantities of alcohol-containing fluids. The extraction of body-warmth through cold drinks the writer considers as very much overrated. He attributes the bad effects of such drinks to irritation of the stomach mucosa, which becomes, therefore, a possible starting-point for acute or chronic inflammatory conditions. In the normal stomach, the author concludes, not only drinking at meals, within certain limits, does not interfere with digestion, it even aids this process. With patients suffering from stomach or other diseases, however, the case is different. Drinking *ad libitum* cannot be allowed. To the question, Shall patients drink nothing with their meals? Dr. Ewald answers that he sees no reason why small amounts of fluid should not be allowed, excepting to patients suffering from dilation of the stomach. As above shown, fluids, and particularly alcoholic and carbonated fluids, will, even in limited amounts, aid digestion and increase appetite, and will more than counterbalance the so-called effects of drinking at meals, viz., the possible slowing of digestion, the dilution of the solid constituents of the meal, the overburdening of the stomach, a very improbable lowering of the body temperature, etc. Even admitting that such effects occur, the question of drinking before, during or after meals Dr. Ewing considers as belonging to the hocus-pocus of suggestion therapy; the physiological act is not influenced if fluid is taken one-half hour sooner or later. The fluid should not be very cold; further, we must follow the indications of the disease and as far as possible the wish of the patient. Naturally, alcoholic fluids that have a direct local irritating effect will be withheld—*e. g.*, in ulcer of the stomach, new inflammatory processes, necrosing neoplasms. Another question is, how far shall we allow abnormally increased thirst to be quenched, as in diabetes, fever, and some chronic diseases? The writer answers that the thirst should be quenched with as little liquid as possible. This is particularly true in case of stomach dilation, when the patients have the tendency to drink large quantities, partly because stomach absorption is very slow and imperfect. Moreover, though this seems paradoxical, thirst may be lessened by forbidding water as a drink. Then, too, thirst very often depends upon dryness of the mouth and pharynx; hence, frequent moistening of the mouth and gargling will often lessen thirst.—*Dietetic and Hygienic Gazette.*

### THE STERNOMASTOID TUMORS OF CHILDREN.

The causation of these little tumors has been the subject of considerable discussion. In these discussions the peculiar tendency of the human mind to seek an explanation for any phenomenon in "the soft atmosphere of mystery" has been clearly apparent. Instead of seeking an explanation based upon mechanical laws and obvious facts, some minds are prone to grope after the strange and mysterious. In the case of these tumors, an explanation based upon mechanics is the only rational one.

They are discovered usually in the second or third week after birth, and as smooth, rounded, hard swellings within the body of the sternomastoid muscle. They can be moved with the muscle from side to side only. They seem rarely to be painful, but when first discovered are in some cases sensitive to touch. The overlying skin is neither adherent nor discolored. There is usually a certain amount of torticollis, the face being turned away from the tumor.

The causation is shown in part by the frequency with which they occur after difficult or instrumental labor—in other words, labor which puts a particular strain upon these muscles. They are especially common after breech presentations, two-thirds of the cases being associated with that condition. The tumor is then upon the right side. When following head presentations it is commonly found on the left side. These facts are clearly explained by the common mechanism of labor.

In a highly interesting paper on wryneck and sternomastoid tumors in children, in a recent number of the *Birmingham Medical Review*, Dr. Jordan Lloyd reports twenty-six cases; of these patients fifteen were boys, eleven were girls. In the case of sixteen the right side was involved, in ten the left side. The author lays special stress upon the importance of these tumors in causing the persistent painless form of wryneck seen in children.

Autopsies upon this condition have not been large in number, but have been sufficient to establish quite positively the histological condition. Spencer found that microscopical sections show clearly that there was rupture of the muscular fibers, with effusion of blood. Witzel has shown that subsequent deformity is caused by the formation of contracting fibrous bands. Delafield and Prudden say that when muscle-fiber is ruptured repair usually takes place by the production of granulation tissue, which gradually becomes converted into cicatricial tissue, thus binding the parts together. Lloyd describes the swelling in the later stages as consisting of bundles of white fibrous tissue, blending gradually with normal adjacent muscle fibers. It consists, in fact, of reparative material in the course of organization, and is thrown out in excess, as is seen in other parts where injured muscles, tendons or bones are not kept at rest during their process of repair. Holt describes the tumors as due partly to blood extravasation and partly to inflammatory products. Like Rotch, Jacobi and Davis, he classifies them under the head of *hematoma.*

These little tumors disappear spontaneously, being rarely seen after the third month. They require no treatment; surgical interference should, above all, be avoided. They are of no serious import, except in the small number of cases in which they are followed by torticollis, due to contraction of the muscle. That condition requires appropriate orthopedic management.—*Archives of Pediatrics.*

---

### THE TONGUE AS A CLINICAL GUIDE IN DISEASE.

A recent number of the *Indian Medical Recorder* gives the following in regard to the tongue:

A broad, pallid tongue, with a loaded base, says atony, and refers you to a want of action of the entire viscera below.

The remedial agents would be cathartics and tonics, especially those mild but effectual in character.

A shrunken tongue, pinched in expression, indicates functional inactivity of digestion, and requires great care in choice of food as well as quantity. In this condition of tongue we have atony also.

It is the tongue of advanced fevers, inflammations of the mucous membranes, and want of assimilation, hence great caution both of remedies and food. Here we must not use cathartics. Mild aperients may be carefully used.

A contracted, pointed tongue, with dryness and dark fur, is the usual tongue of typhoid fever and other low grades of fever, when all thinking minds would use great care in the treatment and food.

The dryness or moisture of the tongue denotes the extent of the disease of the intestines, and will point us in that direction.

A fissured tongue points to the kidneys, either an inflammation or something wrong with secretion.

Yellow coatings are usually associated with morbid liver and want of biliary secretions, and would indicate mild hepatics and tonics.

Raised papillæ, bright red, denote irritation of ganglionic nerves and irritation of stomach, especially the mucous coating. Shows exhaustion; no digestion, and needs rest; nux vomica twenty drops, and the food to be warm and taken in small quantities. Bismuth and pepsin after food.

Broad, thick tongue, papillæ not visible, but looking raw, denotes a septic condition of blood, and favors typhoid fever. Indicates, if deep red, sulphuric acid; if pale, sulphite of soda. Liquid food, sipped warm, in small quantities.

Deep, dark red tongue and dark coating indicate septic condition of blood.

Shades of dark brown and black denote typhoid condition or septic conditions.

Pale, dirty fur on tongue denotes acidity, and a septic condition of system; indicates sulphite of soda; but if membranes are deep red, sulphuric acid will be admissible, because it would show an alkaline condition of blood.

Contracted, pointed, inability to hold still, and drawn to one side of mouth, denotes trouble with the nerves, and perhaps the brain. Requires great care and study of condition.

Dry tongue always denotes feverishness or inflammatory condition, affection of the nerve-centers of ganglia.

Thick tongue, and curved edges upward, denotes atony of the nerve-centers of ganglia, requiring stimulants, nux vomica or strychnine and quinine.

Pointed, narrow tongue is the tongue of sluggish condition of digestion and assimilation and congestion, especially of the base of brain. Restlessness and constant change of position are usually present.—*Medical Age.*

---

### THE FORAMEN OF WINSLOW.

Desirous of aiding as far as possible all medical students who are now about undergoing the severe trials of memory known as first- and second-year examinations in anatomy, we publish the following poetic and at the same time accurate description of an important and difficult anatomical region—one, in fact, which may be described as the central point about which abdominal anatomy is grouped, or as the key to the situation. With regard to this fact the following dictum has been attributed to Hippocrates: "The way to *learn* anatomy is to begin at the foramen of Winslow and work outward."

Combining, as they do, accurate anatomical description with the true lyric touch, these verses, which have lately been discovered unsigned among some old papers, have by some been attributed to our own poet and anatomist, Oliver Wendell Holmes, although this theory of their authorship has not been fully confirmed.

### THE FORAMEN'S LAMENT.

I'm a poor abused foramen,
After Winslow I am named,
As a sticker and a poser
I am most unjustly famed.
For the students, all neglecting,
Often fail in their detecting,
While my owner they're dissecting,
Then most roundly am I blamed.

I'm a poor abused foramen,
And in front of me is tucked
The communis choledochus, which
Is nothing but a duct.
Farther fr[illegible]nt the duodenum—
Hepatic artery between 'em—
And the portal vein, you've seen 'em—
But if not you're surely plucked.

I'm a poor abused foramen,
And I'm bounded on the back
By the lower vena cava,
For I'm just before its track,

The right crus of the diaphragm
Then helps show you where I am,
As I guide you from the greater
Over to the lesser sac.

I'm but a poor foramen,
And the lobe Spigelii
Is kind enough to locate so's
To form the top of me;
The vessel that I've named before
Is kind enough to form the floor,
Just he alone and nothing more,
Hepatic artery is he.

Now, gentle reader, listen,
Have I not good cause for sadness?
My descriptions never tally, and
They drive me most to madness
As I read the verbal photographs
That pass from book to book,
And make me out the darndest thing
That ever looked a look.
I know I don't deserve it,
I'm a simple little hole,
And the thought of these descriptions
Harrows up my very soul.

—*Boston Medical and Surgical Journal.*

---

### REMOVAL OF ENTIRE STOMACH.

In the *Boston Medical and Surgical Journal*, May 5, Brigham, of San Francisco, reports in detail a "Case of Removal of the Entire Stomach for Carcinoma; Successful Esophago-duodenostomy; Recovery." The operation was performed on a woman, aged sixty-six years, who had complained for the past year. She had, however, been able to digest her food until Christmas time, when she vomited any solid food taken. The operation was done February 28, lasted two hours and a quarter, and with a loss of blood of but two ounces. He says: "In the treatment of the case no attempt had been made to predigest the nourishment which was given to the patient. The precaution was taken, however, to supply easily digested food, and when meat was allowed it was cut in very small pieces. The food was taken slowly, whether liquid or solid. It is no hardship for the patient to live on simple food, for she has done so all her life; and especially, as age has advanced, she has been obliged to eat food that required the least chewing. The food was given of medium temperature; water was taken as it came from the pipe, and wine as it stood in the room; iced cream, of which the patient was particularly fond, was taken slowly so that it dissolved in the mouth before it was swallowed. At first everything was too salt; as the patient got well she wished salt on both eggs and oysters. The amount of flatus in the bowels was enough to cause pain only a few times in the early part of her illness. The urine has been normal throughout. Never since the operation has any undigested food been seen in the movements from the bowels, and for the most part these have been wholly or partly formed. The patient has vomited but a few times since the operation—twice after etherizations, twice after some laxative had been given, once after the button left its place, and twice after coughing; not more than six ounces at any one time, generally much less. On three or four occasions a mouthful of food would be regurgitated—an oyster, some shreds of meat, or a few teaspoonfuls of coffee. As a usual thing the food was well retained and well digested. Milk, which would sustain most patients under such circumstances, was not liked, and an important food was thus unavailable. The patient's skin is in a natural condition, without any dryness; this may be due to the thorough washing which the entire body has had daily since the operation. The symptom which gave the most anxiety after the operation was the restlessness, which was unusually marked. This was without doubt the result of the surgical shock which was caused by the removal of so important an organ as the stomach, and the interfering with its vessels and nerves. The season of the year in California, with mild, sunny days, and the careful and constant nursing, are among the factors which made the operation a success. The age of the patient counted for something also; the effects of the change of life had long passed by, and there had been for many years an even condition of good health. . . She has a fine color; complains of nothing so far as the functions of her body go; eats whatever she wishes; has no pain whatever; is of a very cheerful disposition. She is out of doors most of the day from 10 till 5 o'clock, taking occasional walks around the hospital grounds; her temperature and pulse are normal; she sleeps without an opiate. Although she has food every three hours she feels hungry at times, and feels that she could eat twice as much as is given to her. She is gaining in weight, and her general condition at the present time, April 14, seven weeks after the operation, is satisfactory in every respect."—*Jour. Am. Med. Asso.*

---

### PRESCRIPTIONS.

### VOMITING OF PREGNANCY.

℞ Ingluvin ........................ gr. xl.
Cerei........................ gr. x.
Pulveris ipecacuanhæ........................ gr. v.
Creosoti ........................ m. v.
M. et div. in chart. No. 10.
Sig. One powder taken every hour until nausea is controlled.

### CATARRHAL JAUNDICE.

℞ Ammonii iodidi........................ ʒ i.

Liq. potassii arsenitis........................ ʒ ss.

Tincturæ calumbæ ........................ ʒ iv.

Aquæ destil........................ ℥ iss.

M. Sig. Teaspoonful before meals.—*Bartholow.*

### MENORRHAGIA.

℞ Acidi gallici ........................ gr. xv.
Acidi sulphurici aromat ........................ m. xv.
Tincturæ cinamomi ........................ ʒ ii.
Aquæ destil........................ ℥ ii.
M. Sig. One dose; take every four hours until bleeding ceases. (In profuse bleeding.)—*Hazard.*

### AMENORRHEA.

℞ Tincturæ ferri chloridi........................ ʒ iii.

Tincturæ cantharidis........................ ʒ i.

Tincturæ guaiaci ammoniatæ........................ ℥ iss.

Tincturæ aloes........................ ʒ iv.

Syrupi simplicis........................ q. s. ad. ℥ vi.

M. Sig. Tablespoonful three times a day.—*Dewees.*

℞ Extracti aloes........................ ʒ i.

Ferri sulphatis exsiccati........................ ʒ ii.

Asafœtidæ........................ ʒ iv

M. ft. pil. No. 100.

Sig. One pill three times a day.—*Goddell.*

### DELIRIUM TREMENS.

℞ Potassii bromidi ℥ i.

Chloralis ℨ iv.

Tincturæ digitalis ℨ i.

Tincturæ capsici ℨ i.

Tincturæ zingiberis ℨ i.

Spiritus ammoniæ aromatici ℨ i.

Syrupi aurantii cortici ℨ i.

Aquæ q. s. ad. ℥ viii.

M. Sig. Dose, a tablespoonful.—*Bellevue Hospital.*

### ALOPECIA.

℞ Extracti pilocarpi fluidi ℥ i.

Tincturæ cantharidis ℨ iv.

Linimenti saponis q. s. ad. ℥ iv.

M. Sig. Rub into the scalp daily.—*Bartholow.*

### INTERCOSTAL NEURALGIA.

℞ Chloralis,
Camphoræ, } aa ℨ i.
Mentholis,
M. Sig. With a brush spread a layer of this mixture (which is liquid) over the painful parts. Renew the application when the pain reappears.—*Dr. S. Solis-Cohen.*

### PAINLESS BLISTER.

℞ Mentholis gr. xx.
Chloralis gr. xx.
Olei theobromatis gr. xxx.
Spermaceti ℨ i.
M. Make into a paste.—*La Médecine Moderne.*

### LOCAL ANESTHETIC.

℞ Mentholis ℨ i.

Chloroformi ℨ x.

Ætheris ℨ xv.

M. Sig. Use as a spray over field of operation. Anesthesia lasts from two to six minutes.—*Louisville Medical Monthly.*

### VULVITIS.

℞ Liquoris plumbi subacetatis ℨ i.

Tincturæ hyoscyami ℨ ii.

Aquæ camphoræ q. s. ad. ℥ viii.

M. et. ft. lotio. Sig. Apply constantly, tepid, with saturated cloth.—*Waring.*

### RHEUMATISM—CHRONIC.

℞ Liquoris potassii arsenitis ℨ ii.

Potassii iodidi ℥ ii.

Syrupi simplicis ℥ iii.

M. Sig. Teaspoonful three times a day in water, between meals.—*De Costa.*

(In the treatment of rheumatism, whether acute or chronic, vegetable cholagogues, such as enonymin, leptandrin, podophyllin, etc., should be daily administered along with other anti-rheumatic remedies.—Editor.)—*Medical Standard.*

### PTYALISM.

℞ Potassii chloratis ........ gr. xvi.
Tincturæ ferri chloridi ........ ʒ iiss.
Glycerini ........ ℥ i.
Aquæ ........ q. s. ad. ℥ ii.
M. Sig. Teaspoonful in water every two hours.

### LUMBAGO.

℞ Potassii iodidi ........ ʒ ss.

Tincturæ opii deod ........ ʒ ii.

Spiritus lavandulæ comp ........ ʒ i.

Spiritus ætheris nitrosi ........ ℥ ss.

Aquæ destillatæ ........ ℥ xii.

M. Sig. Take two tablespoonfuls twice daily.—*Sir B. Brodie.*

### GOUT.

℞ Tincturæ colchici seminis ........ m. xv.
Magnesii carbonatis ........ gr. vi.
Magnesii sulphatis ........ gr. xxx.
Aquæ menthæ piperitæ ........ q. s. ad. ℥ i.
Fiat haustus.
Sig. Repeat according to circumstances.—*University Hospital.*

### ASTHMA.

℞ Potassii iodidi ........ ʒ iii.

Extracti belladonnæ fluidi ........ ʒ i.

Extracti lobeliæ fluidi ........ ʒ ii.

Extracti grindeliæ fluidi ........ ʒ iv.

Glycerini, Aquæ destillatæ, } aa ........ ℥ iss.

M. Sig. Take a teaspoonful every two, three or four hours, as necessary.—*Bartholow.*

### ANTISEPTIC FLUID (SEILER'S).

℞ Thymol, Eucalyptol } aa ........ gr. x.

Menthol ........ gr. v.

Olei gaultheriæ ........ gtt. vi.

Sodii benzoati, Sodii boratis, Sodii salicylatis } aa ........ gr. xx.

Glycerini ........ ℥ ss.

Alcoholis ........ ℥ i.

Aquæ ........ q. s. ad. ℥ viii.

### ANTISEPTIC FLUID COMP. (SEILER'S).

℞ Antiseptic fluid ........ ℥ i.

Sodii bicarbonatis, Sodii boratis } aa ........ ʒ i.

Glycerini ........ ℥ i.

Aquæ ........ q. s. ad. ℥ iv.

M. Sig. Add to one quart of water and use as a disinfectant wash.—*Ex.*

